# Cost-effectiveness analysis of sodium zirconium cyclosilicate for treating hyperkalemia among Chinese patients

**DOI:** 10.3389/fpubh.2023.1196789

**Published:** 2023-12-07

**Authors:** Lei Tian, Shihui Fu, Mengyuan Li, Xinrui Zhao, Hongchao Li

**Affiliations:** School of International Pharmaceutical Business, China Pharmaceutical University, Nanjing, China

**Keywords:** hyperkalemia, sodium zirconium cyclosilicate, chronic kidney disease, heart failure, China, cost-effectiveness

## Abstract

**Objectives:**

Hyperkalemia most commonly develops in chronic kidney disease (CKD) or heart failure (HF) patients. Sodium zirconium cyclosilicate (SZC) is a new selective potassium (K+) binder for treating hyperkalemia. The aim of this study was to evaluate the cost-effectiveness of SZC vs. usual care for the treatment of hyperkalemia among CKD patients or HF patients in China.

**Methods:**

Individual patient microsimulation models were constructed to simulate a CKD cohort until the initiation of renal replacement therapy (RRT) and a HF cohort across the lifetime horizon. K+ levels were based on two phase 3 clinical trials. Health state utility and event incidence rates were retrieved from literature. Drug costs and healthcare utilization costs were obtained from negotiated price, literature, and expert interviews. Costs and quality-adjusted life-years (QALYs) were both discounted at 5%. The main outcomes were overall costs, QALYs, and incremental cost-effectiveness ratio (ICER). The willingness-to-pay (WTP) threshold in China is CNY 80,976-242,928/QALY, which is one to three times the gross domestic product *per capita*. Sensitivity analyses were performed to characterize the models’ uncertainty.

**Results:**

In the HF cohort, the base case results revealed that SZC was associated with 2.86 QALYs and the total cost was CNY 92671.58; usual care was associated with 1.81 QALYs and CNY 54101.26. In the CKD cohort, SZC was associated with 3.23 QALYs and CNY 121416.82 total cost; usual care was associated with 2.91 QALYs and CNY 111464.57. SZC resulted in an ICER of CNY 36735.87/QALY for the HF cohort and CNY 31181.55/QALY for the CKD cohort, respectively. The one-way and probability sensitivity analyses found that the results were robust.

**Conclusion:**

SZC is a cost-effective treatment compared to usual care in HF and CKD patients. SZC is an important novel treatment option for managing patients with hyperkalemia in China.

## 1 Introduction

Hyperkalemia refers to an elevated concentration of potassium (K+) in the serum (typically defined as >5.5 mmol/L), and develops when there is excessive production or ineffective elimination of K+ (1, 2). Elevated serum potassium leads to hyperkalemia and an increased risk of hospitalization, cardiovascular disease, and death (2–4). Observational studies have shown that patients with CKD and HF are more likely to develop hyperkalemia than the general population (5). Renin-angiotensin-aldosterone system inhibitors (RAASi) are an important therapeutic strategy for CKD and HF patients, as RAAS plays a crucial role in the regulation of blood volume, blood pressure (BP), and cardiovascular function (6, 7). However, RAASi can exacerbate hyperkalemia, with adverse outcomes compounding the risk of hyperkalemia (8–11).

Epidemiological survey results show that the incidence of CKD in Chinese adults is 10.8%, and the number of patients is estimated to be 119.5 million (12). In the CKD patients requiring hemodialysis, 14.64% of them are complicated with hyperkalemia (13). An epidemiological survey conducted in 157 hospitals in China showed that in patients with chronic kidney disease and heart failure, the rates of patients who experienced hyperkalemia were 22.89% and 12.54% (14). The incidence of hyperkalemia varies significantly between stages of CKD. According to the Hospital of DaLian Medical University data from 2011 to 2015, the incidence of CKD 3–5 hyperkalemia was 4.60%, 9.33%, and 18.39%, respectively (15).

The current usual care for hyperkalemia in China mainly combines the basic treatment strategy, including insulin, glucose, calcium gluconate and furosemide combined with calcium polystyrene sulfonate, but the strategy is only suitable for short-term treatment. There has been controversy regarding calcium polystyrene (CPS) sulfonate efficacy and safety, the use of it for a long-term may lead to colonic necrosis (CN) (16, 17). In current clinical practice, no long-term serum potassium management is carried out after the serum potassium level returns to normal besides lifestyle interventions. In recent years, two novel drugs for hyperkalemia, patiromer and sodium zirconium cyclosilicate (SZC), were approved in the United States and European Union, but only SZC has been marketed in China. SZC is a new highly selective oral cation exchanger that binds K+ in the gastrointestinal tract, and may subsequently be used to lower serum K+ levels in patients with hyperkalemia (18, 19). The efficacy and safety of SZC has been assessed in two phase clinical trials (ZS-004 and ZS-005) investigating the acute treatment of hyperkalemia, and the ongoing maintenance normokalemia (20–22). In the ZS-005 trial, results found that 99% of participants treated with SZC achieved normokalaemia during the acute phase (treatment with 10 g SZC, three times daily for 24 to 72 h), and entered the maintenance and long-term management phase of 10 g SZC per day. At the maintenance phase, the mean serum potassium level was 4.8 mmol/L (95%CI, 4.7 to 4.8). The results showed that SZC administration was associated with rapid correction of hyperkalemia and long-term maintenance of normokalemia (20).

There has been no study to evaluate the health economic outcomes of the SZC in patients from the perspective of the Chinese healthcare system. This study aimed to conduct a cost-effectiveness analysis of a treatment strategy including sodium zirconium cyclosilicate vs. usual care for treating hyperkalemia among CKD patients and HF patients in China.

## 2 Methods

### 2.1 Overview

An individual patient microsimulation model was constructed in Microsoft Excel to evaluate the cost-effectiveness of SZC vs. usual care for treating hyperkalemia among CKD patients and HF patients from the perspective of the Chinese healthcare system. Patient-level data from two large international clinical trials (ZS-004 and ZS-005) of SZC for hyperkalemia were used to define the baseline distribution of patients according to age, sex and disease severity. The simulation cohorts comprised 63.56-year-old CKD patients with an estimated glomerular filtration rate (eGFR) of 31.63 mL/min/1.73 m^2^ and 65.07-year-old HF patients with eGFR of 68.14 mL/min/1.73 m^2^ at baseline. In both cohorts, the proportion of male and receiving RAASi were 63% and 70.20% (23, 24). Costs and benefits were discounted at 5% per year (25). Model outputs cost, life years (LYs), quality-adjusted life years (QALYs) and incremental cost–benefit ratio (ICER; Incremental cost/incremental QALYs).

### 2.2 Model structure

**Figure 1:**
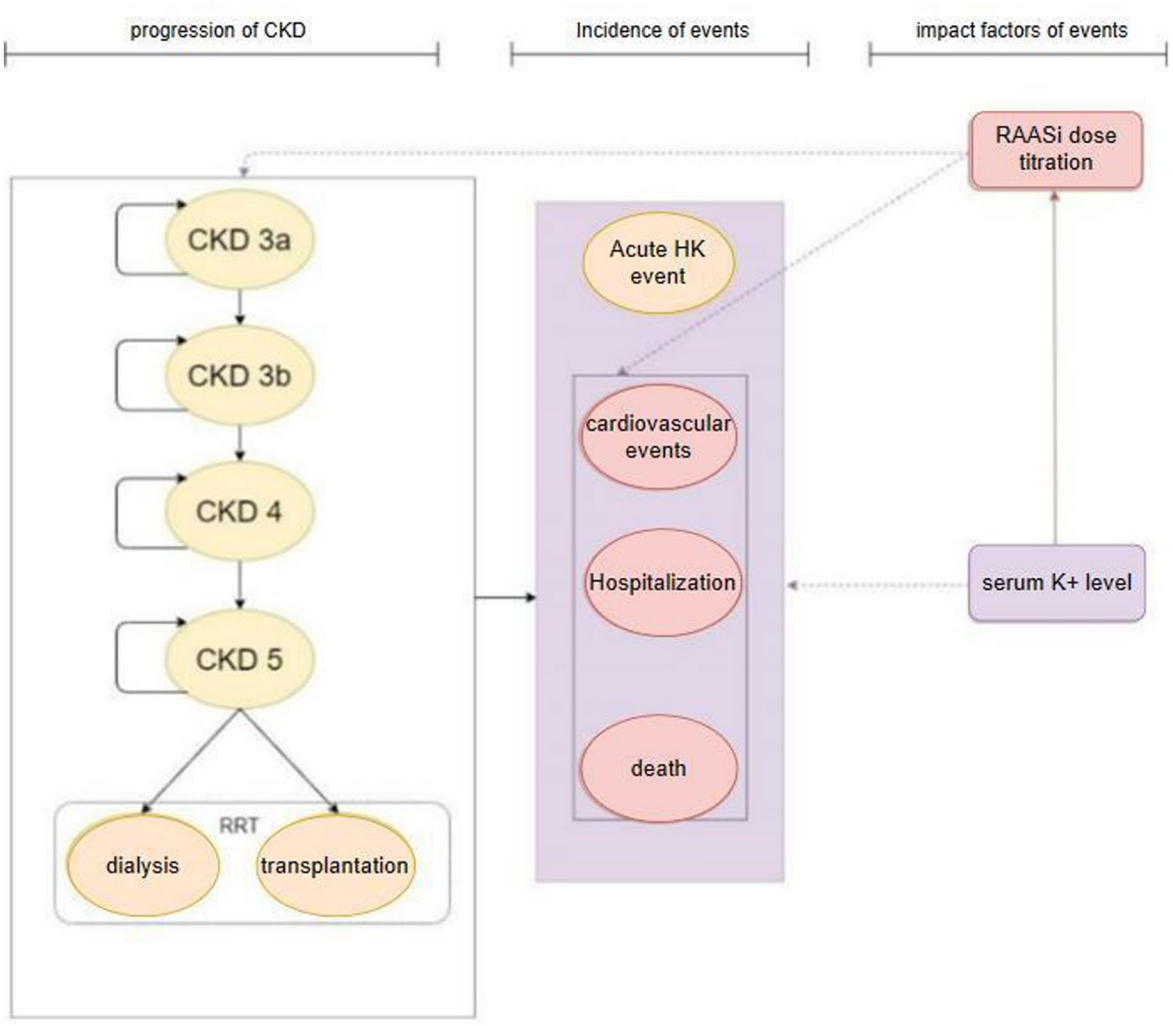
Model structure: CKD cohort. CKD, chronic kidney disease; HK event, hyperkalemia event; RRT, renal replacement therapy; RAASi, renin-angiotensin-aldosterone system inhibitors.

**Figure 2:**
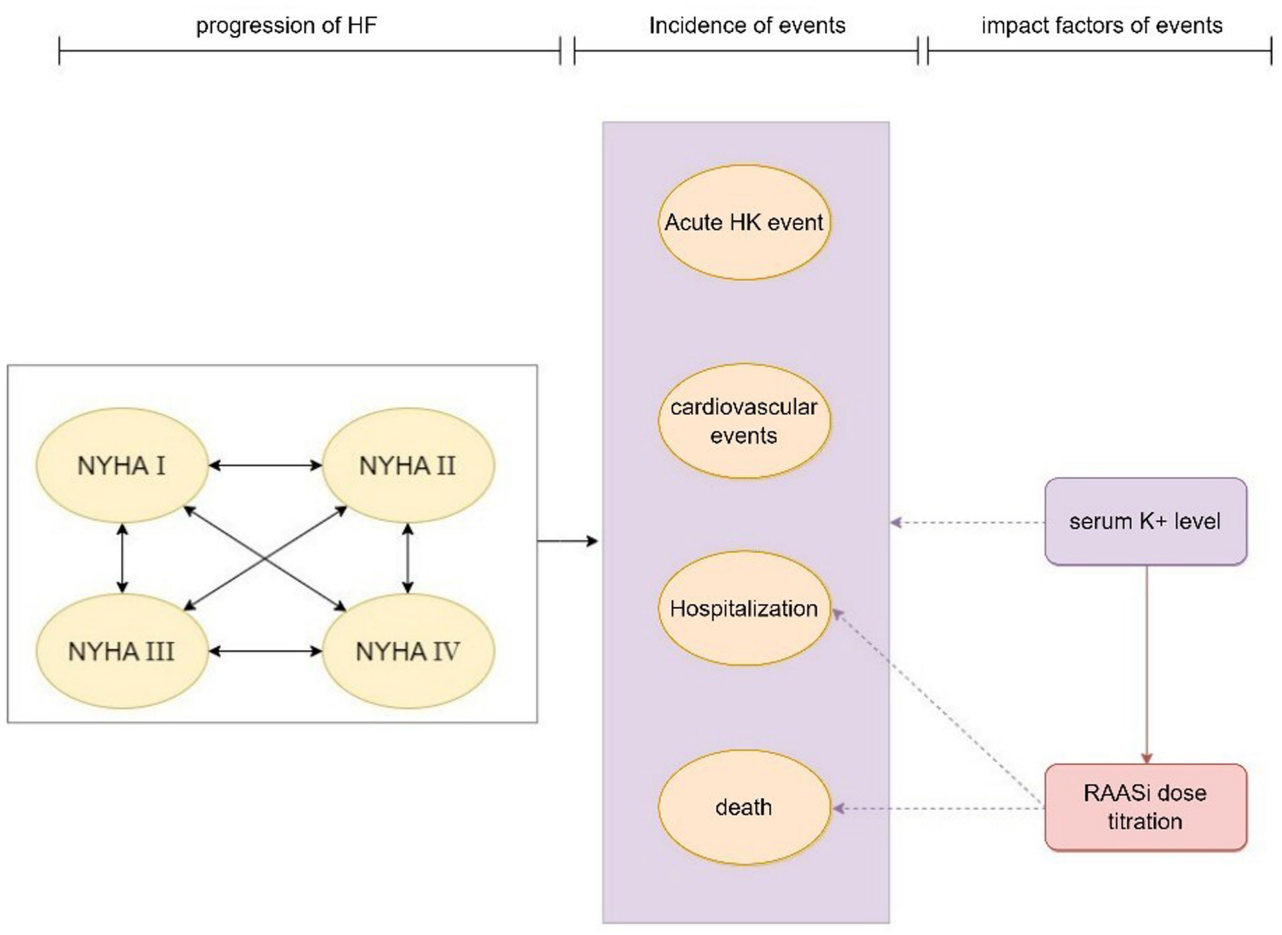
Model structure: HF cohort. HF, heart failure; HK event, hyperkalemia event; NYHA, New York Heart Association; RAASi, renin-angiotensin-aldosterone system inhibitors.

The model’s primary cycle length was 4 weeks (28 days), and the serum potassium level were simulated in each cycle. The model for the CKD cohort included 6 CKD-related health states: CKD 3a, CKD 3b, CKD 4, CKD 5, renal replacement therapy (dialysis, kidney transplantation), death (Figure 1). For the simulation, all patients in the cohort initiated with CKD 3a according to the base eGFR, the progression of CKD patients was modeled via the annual decline rate of eGFR, and progression through CKD stages were tracked until the onset of end-stage renal disease (ESRD) and the initiation of renal replacement therapy (RRT). The management of K+ following the initiation of RRT differs from that prior to ESRD. Therefore, patients would not be modeled after RRT. The model used in the HF cohort included five HF related health states according to New York Heart Association (NYHA) Class: NYHA I, NYHA II, NYHA III, NYHA IV, death (Figure 2). Patients in the simulated cohort were placed in different NYHA classes according to set proportions. Patient progression was modeled by transitions between NYHA classes (I to IV). In both cohorts, model had the same complications: acute hyperkalemia events, hospitalization, and cardiovascular events. Death was an absorption state.

Modeled serum K+ parameters were treatment-dependent, and the incidence of acute hyperkalemia was triggered when modeled K+ levels exceeded a threshold once in 28 days. Three K+ thresholds (high: 6.5 mmol/L, mid: 6.0 mmol/L, low: 5.5 mmol/L) were specified to define the severity of the acute hyperkalemia event (mild, moderate and severe acute hyperkalemia event), and to model differing management strategies include basic treatment for hyperkalemia event and proportion of RAASi down-titrated or discontinued based on event severity. The probability of other events (including RAASi dosage titration, hospitalization, cardiovascular events, and death) were modified by serum K+ levels and RAASi dosage in use.

### 2.3 Treatment strategies

The usual care for hyperkalemia in China was determined by expert interviews and used as a comparison for this study, therefore the control arm was comprised of CPS combined with conventional basic treatment (insulin, glucose, calcium gluconate and furosemide) during the acute phase of a hyperkalemia event, followed by lifestyle intervention during the long-term management. For the intervention arm, as the 28-day duration of SZC in long-term management phase is relatively consistent with the actual clinical situation in China, patients received 28 days SZC as long-term management, combined with acute conventional basic treatment. All patients discontinue treatment in the next model cycle unless the simulated serum K+ value reaches the threshold of 5.5 mmol/L. The discontinued patient will begin repeated treatment after the next 28-day cycle. The annual discontinuation probability of SZC was 37.5% from the extended phase of the ZS-005 trial (20). The discontinuation probability of control arm was assumed to be 0. Once the patients treated with SZC stopped taking the drug, they were treated as the control arm.

### 2.4 Model inputs

#### 2.4.1 Progression of disease

The disease progression of patients with CKD was divided into stage 3a(eGFR 45~60), stage 3b(eGFR 30~45), stage 4(eGFR 15~30), and stage 5(eGFR 0~15) according to eGFR, and the natural history evolution of renal function was modeled via a linear decline in eGFR. The annual decline rate of eGFR was obtained from the literature. eGFR in patients using RAASi drugs decreased by 2.34 mL/min/1.73 m^2^ per year, and in patients disusing RAASi drugs decreased by 3.52 mL/min/1.73 m^2^ per year (26). The progression of CKD to ESRD and the subsequent initiation of RRT was modeled according to declining eGFR on a continuous scale, and RRT was initiated once eGFR falls below 8.6 mL/min/1.73 m^2^ (27). In HF cohort, the proportion from NYHA I to NYHA IV was 23%, 48%, 25%, and 4% (28). The monthly transition probability between NYHA I and NYHA IV was obtained from the literature (Supplementary Table S1) (29). It is assumed that the use of RAASi medication does not affect state transition in HF population.

#### 2.4.2 Modeling serum K+ level and RAASi dose titration

In order to better simulate the change level of serum potassium, patients’ serum potassium levels were simulated at each cycle, and mixed-effects regression models using individual patient data obtained from the ZS-004 and ZS-005 trials were used to simulate the changes in serum potassium levels in patients with different baseline serum potassium levels (5.5 mmol/L or 6.0 mmol/L). K+ levels fluctuate over time, with each patient exhibiting a unique K+ trajectory. To reflect this within the model, treatment- and patient-specific K+ profiles were simulated using mixed-effects regression models (Supplementary Table S2). In ZS-004, both groups received SZC treatment in the acute stage (21, 22). We conservatively assumed that the effect of usual care was the same as SZC, through using pooled data of both arms using SZC in ZS-004 trial, a sets of estimates for the acute phase produced, which simulate K+ level of intervention arm and control arm in the model. Over the post-acute phase (day 4 onwards), serum K+ profiles were modeled using the placebo arm of ZS-004 for control arm. ZS-005 is a single-arm clinical trial, all patients were treated with SZC, considering the large sample size and its representativeness, K+ profiles of intervention arm after acute phase were modeled using pooled data of SZC arm of ZS-004 and ZS-005 trials (20).

RAASi initiation was not modeled in patients not taking RAASi at baseline, while those patients taking RAASi at baseline were assumed to receive the maximum appropriate RAASi dose. Patients were checked for serum potassium levels once a model cycle, according to the expert’s advice to halve the dose or discontinue RAASi if serum potassium levels were above a certain range. Down-titration to a sub-maximum dose, or discontinuation of RAASi treatment (from any dose) may occur, where the proportion of down-titrated or discontinued patients was defined as a function of K+ and derived from Epstein et al. (30) (Supplementary Table S3). To reflect the impermanent nature of these RAASi treatment changes in clinical practice, a return to maximum RAASi use (up-titration) may be modeled for a 49.7% of patients discontinued RAASi after 42.29 weeks. These data were drawn from Luo et al. (31), and we assumed that the proportion and specified duration of time in patients with sub-maximum dose were equal to discontinuation parameters.

#### 2.4.3 Incidence of events

Data used to inform modeled baseline event incidence were primarily sourced from the published literature on the rate of cardiovascular events, hospitalization, and all cause death, and baseline rates were modified according to RAASi use and K+ levels, and the estimated rate of each event was converted to the probability per cycle.

The baseline annual probability of hospitalization, cardiovascular events and death with different stages of CKD were obtained from retrospective studies of patients with CKD, the annual probability were presented in Table 1 (32–34). Incidence rate ratio (IRR) with different serum potassium levels, presented in Supplementary Table S4, were obtained from large electronic health record data (31). The odds ratio (OR) and hazard ratio (HR) of cardiovascular events and death in patients with the highest dose of RAASi compared with patients without RAASi were 0.82 and 0.87, respectively, which were obtained from the published meta-analysis (43). We assumed that dose was proportional to efficacy, and calculate the OR of 0.91 for cardiovascular events and HR of 0.93 for death among patients who used highest dose of RAASi as compared with those who discontinued RAASi. Since there is no evidence found to support the use of RAASi can reduce the risk of hospital admissions, we assumed no effect of RAASi treatment on hospitalization.

**Table 1:** Parameters used in the cost-effectiveness analysis.

Input parameters	Values	Distributions	Standard Error (SE)	Sources
**Annual probability of hospitalization**				
CKD 3a	0.1722	Normal	0.0045	Go et al. (32)
CKD 3b	0.4526	Normal	0.0067	Go et al. (32)
CKD 4	0.8675	Normal	0.0090	Go et al. (32)
CKD 5	1.4461	Normal	0.0090	Go et al. (32)
**Annual probability of cardiovascular events**				
CKD 3a	0.0365	Normal	0.0012	Go et al. (32)
CKD 3b	0.1129	Normal	0.0012	Go et al. (32)
CKD 4	0.2180	Normal	0.0024	Go et al. (32)
CKD 5	0.3660	Normal	0.0048	Go et al. (32)
**Annual mortality**				
CKD 3a	0.0241	Normal	0.0004	Liu et al. (33)
CKD 3b	0.0241	Normal	0.0007	Liu et al. (33)
CKD 4	0.1136	Normal	0.0018	Go et al. (32)
CKD 5	0.1521	Normal	0.0031	Tseng et al. (34)
**Utilities**				
Health state utility: CKD 3a	0.8400	Beta	0.0840	Go et al. (35)
Health state utility: CKD 3b	0.8400	Beta	0.0840	Go et al. (35)
Health state utility: CKD 4	0.7700	Beta	0.0770	Go et al. (35)
Health state utility: CKD 5	0.6500	Beta	0.0650	Go et al. (35)
Health state utility: NYHA I	0.7300	Beta	0.0730	Xuan et al. (36)
Health state utility: NYHA II	0.7800	Beta	0.0780	Xuan et al. (36)
Health state utility: NYHA III	0.7200	Beta	0.0720	Xuan et al. (36)
Health state utility: NYHA IV	0.6600	Beta	0.0660	Xuan et al. (36)
Disutility: cardiovascular events	−0.0500	Beta	0.0400	Kent et al. (37)
Disutility: hospitalization	−0.0200	Beta	0.0079	Gohler et al. (38)
**Costs (CNY)**				
Total cost of SZC after an acute hyperkalemia event	1026.59	Gamma	104.75	Negotiated price
Total cost of CPS after an acute hyperkalemia event	330.67	Gamma	33.74	Market price
Mild acute hyperkalemia event management costs (K^+^ 5.5–6.0)	403.00	Gamma	41.12	Expert Opinion; Zhang et al. (39)
Moderate acute hyperkalemia event management costs (K^+^ 6.0–6.5)	2672.38	Gamma	272.69	Expert Opinion; Zhang et al. (39)
Severe acute hyperkalemia event management costs (K^+^ > 6.5)	7404.00	Gamma	755.51	Expert Opinion; Zhang et al. (39)
Annual cost of RAASi: optimal therapy (max)	704.93	Gamma	71.93	Market price
Annual cost of RAASi: sub-optimal therapy (sub-max)	352.47	Gamma	35.97	Market price
Event cost: RAASi dose titration	90.54	Gamma	9.24	Expert opinion
Event cost: cardiovascular events	13361.00	Gamma	1363.37	Ma et al. (40)
Event cost: hospitalization (CKD)	11041.80	Gamma	1126.71	China Health Statistics Yearbook (41)
Event cost: hospitalization (HF)	8900.09	Gamma	908.17	China Health Statistics Yearbook (41)
Annual cost CKD 3a	2437.00	Gamma	248.69	Ma et al. (40)
Annual cost CKD 3b	2578.00	Gamma	263.06	Ma et al. (40)
Annual cost CKD 4	3585.00	Gamma	365.79	Ma et al. (40)
Annual cost CKD 5 (pre-RRT)	6836.00	Gamma	697.57	Ma et al. (40)
Annual cost NYHA I	651.00	Gamma	66.43	Ford et al. (42)
Annual cost NYHA II	752.00	Gamma	76.73	Ford et al. (42)
Annual cost NYHA III	975.00	Gamma	99.49	Ford et al. (42)
Annual cost NYHA IV	1041.00	Gamma	106.22	Ford et al. (42)

CKD, chronic kidney disease; HF, heart failure; RAASi, renin-angiotensin-aldosterone system inhibitors; SZC, sodium zirconium cyclosilicate; NYHA, New York Heart Association.

Annual mortality rate per cycle was calculated based on the Seattle Heart Failure Model (44) and baseline data of patients, parameters of the heart failure model and other baseline information of patients with heart failure were shown in Supplementary Tables S5, S6. Hazard ratio (HR) of mortality at different serum potassium levels was derived from published database data studies (Supplementary Table S7) (45). The risk equation was used to model cardiovascular event incidence in HF patients from a retrospective study (Supplementary Table S8) (46). Probability of hospitalization events in HF patients with different serum potassium levels and doses of RAASi were shown in Supplementary Table S9, parameters was derived from a retrospective cohort study, which showed that blood potassium levels and RAASi use has an impact on the probability of hospitalization events in HF patients with heart failure (46, 47).

#### 2.4.4 Utilities

We used quality adjusted life years (QALYs) as a measure of health benefits by multiplying the time spent in each health state by the utility associated with health status. The utility value of the dead state is usually 0, and the utility value of full health is usually 1. The utility for different health states of CKD stage (pre RRT) were derived from a survey of Korean CKD, which measured utility values through standard gamble method (35). The utility for different NYHA grades in HF patients were derived from a quality of life study based on a Chinese HF population (36). Utilities decrement associated with hospitalization events was applied only in the cycle in which the hospitalization occurred (37), utilities decrement associated with cardiovascular event was applied for the first year after the event (38). All the utilities were presented in Table 1.

#### 2.4.5 Costs

As the model considered costs from the perspective of Chinese healthcare system, only direct medical costs were taken into consideration, which included the drug cost of SZC and CPS during acute and management phases, acute hyperkalemia event management costs (conventional basic treatment and medical services costs), annual outpatient costs, RAASi therapy costs, hospitalization and cardiovascular events costs. We used negotiated price and market price for drug costs, and determine the cost according to the dosage in clinical trial and usage in the instructions (20). The costs of acute hyperkalemia event management were referred to clinical expert opinions and published studies (39). The annual outpatient costs of CKD were derived from an economic burden study of Chinese patients and adjusted for the costs based on the increase in outpatients costs for CKD patients in the UK at each stage (40). Outpatient costs for HF patients were derived from an Australian study and converted to 2021 costs in China (42). The annual cost of using RAASi drugs at maximum and sub-maximum doses was calculated by selecting the RAASi drugs that were commonly used and have been included in the National Reimbursement Drug List. According to the expert interview, electrolyte examination is needed to observe the change of serum potassium level when adjusting the dose of RAASi, so we included the cost of diagnosis and examination in analysis. Hospitalization and cardiovascular event costs were derived from published studies in China (Table 1).

### 2.5 Cost-effectiveness analyses

In base case analyses, QALYs gained and overall cost for a model time horizon were used to evaluate the incremental cost-effectiveness ratio (ICER) of two treatment strategies. Conventionally, one to three times the Gross Domestic Product *per capita* (GDP) is recommended as the threshold of willingness-to-pay (WTP), which was CNY 80,976–242,928 in China in 2021. A treatment strategy was considered cost-effective if the ICER was less than the WTP threshold.

Considering the influence of parameter uncertainty on the ICER of the economic evaluation, we conducted deterministic sensitivity analysis (DSA) and probabilistic sensitivity analysis (PSA) to evaluate the robustness of the model results. In the DSA, parameters were varied within a range determined by either published. If data of range were not available, costs were varied by ±20% of the corresponding base case value; and other parameters varied by ±10% of the corresponding base case value. As the cohort was simulated until the initiation of RRT state or died in base case analysis, the lower value of time horizon was set at 5 years. We generated a tornado diagram ranking impact for each parameter from descending order of impact on the ICER. A probabilistic sensitivity analysis was undertaken assigning probability distributions to key model parameters and undertaking Monte-Carlo simulation with 1,000 iterations. Probability and utility parameters were modeled using a beta distribution, rate and risk parameters (IRR, OR, HR) were modeled using a normal distribution, costs were modeled using a gamma distribution.

## 3 Results

Results of the base case analysis, which includes costs, QALYs, life-years gained, and the ICER are presented in Table 2. In CKD cohort, SZC arm generated additional 0.32 QALYs and 0.43 life-years gained per compared with usual care arm, the estimated mean costs for SZC arm were CNY 121416.83 compared with CNY 111464.58 for usual care arm, resulting in an additional cost of CNY 9952.25. Consequently, the ICER was CNY 31181.55/QALY in China, indicating that SZC is projected to be highly cost effective for the treatment of hyperkalemia in CKD patients. In HF cohort, SZC arm generated additional 1.05 QALYs and 1.43 life-years gained per compared with usual care arm, respectively. The estimated mean costs for SZC arm were CNY 92671.58 compared with CNY 54101.26 for usual care arm, resulting in an additional cost of CNY 38570.31, the ICER of SZC in the treatment of hyperkalemia in HF patients was CNY 36735.87/QALY in China compared with usual care.

**Table 2:** Base case results.

	CKD			HF		
	SZC	Usual care	Difference	SZC	Usual care	Difference
Total costs	121416.83	111464.58	9952.25	92671.58	54101.26	38570.31
Total QALYs	3.23	2.91	0.32	2.86	1.81	1.050
Total LYs	4.29	3.86	0.43	3.90	2.47	1.430
ICER	CNY 31181.55/QALY			CNY 36735.87/QALY		

CKD, chronic kidney disease; HF, heart failure; SZC, sodium zirconium cyclosilicate; QALYs, quality-adjusted life-years; LYs, life years; ICER, incremental cost-effectiveness ratio.

**Figure 3:**
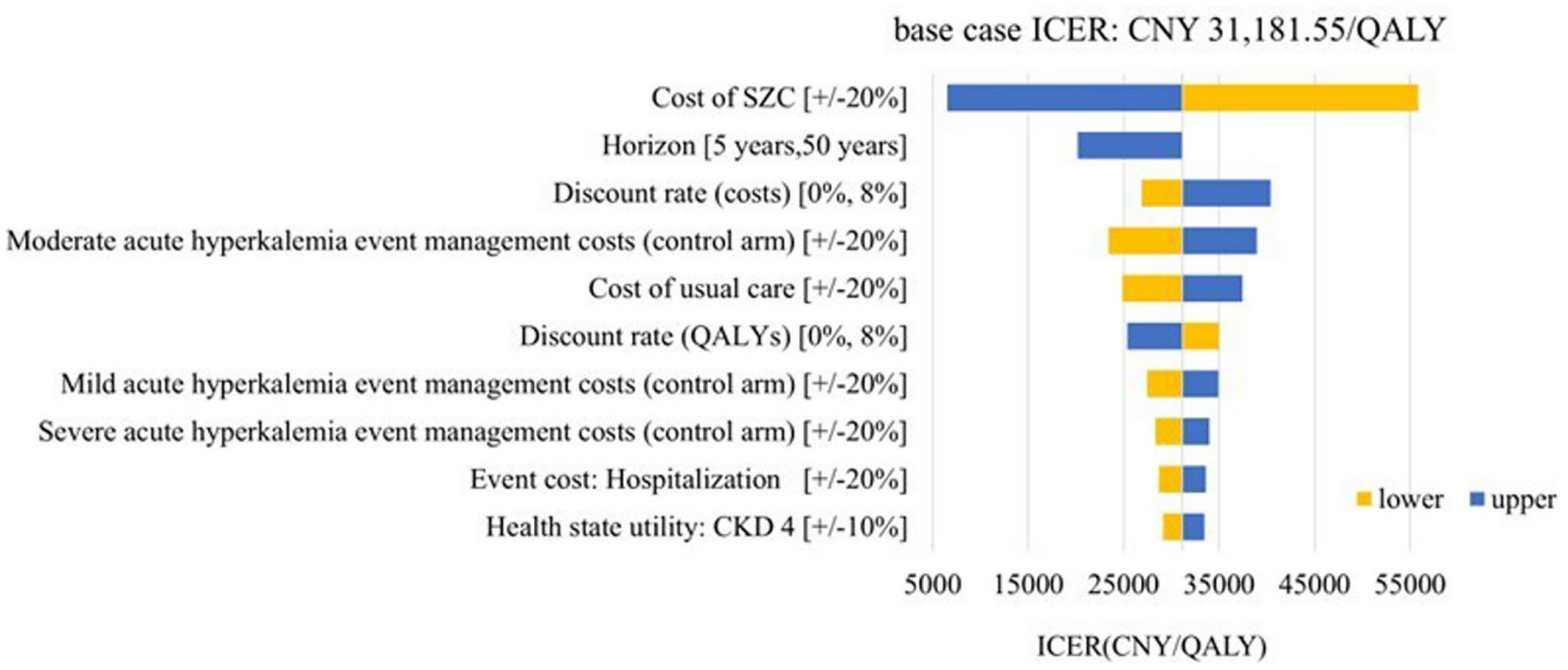
Tornado diagram: CKD cohort. SZC, sodium zirconium cyclosilicate; CKD, chronic kidney disease; eGFR, estimated glomerular filtration rate; ICER, incremental cost-effectiveness ratio; QALYs, quality-adjusted life-years.

**Figure 4:**
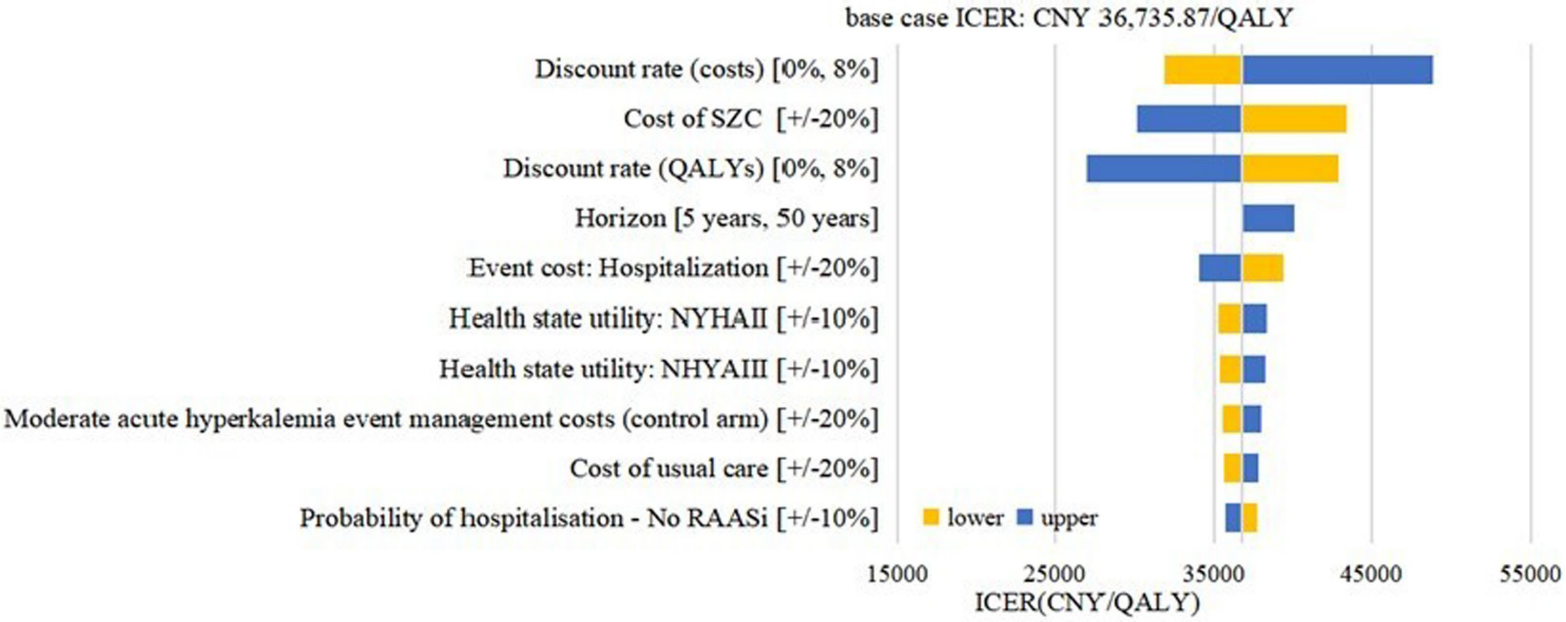
Tornado diagram: HF cohort. SZC, sodium zirconium cyclosilicate; HF, heart failure; RAASi: renin-angiotensin-aldosterone system inhibitors; NYHA, New York Heart Association; ICER, incremental cost-effectiveness ratio; QALYs, quality-adjusted life-years.

Deterministic sensitivity analysis was performed to examine the impact of the assumptions for model inputs on the cost effectiveness. The impact of the 10 most influential parameters was illustrated in Figure 3 (CKD cohort) and Figure 4 (HF cohort). The CKD cohort’s most influential parameter was the cost of SZC (ICER range: CNY 6516.15–55846.96 per QALY gained). In HF cohort, the most influential parameter was the discount rate of the costs (ICER range: CNY 31860.60–48797.32 per QALY gained). However, none of these factors could reverse the base case result within the possible current values yet because the ICERs remained below the conservative cost-effectiveness threshold 1 time GDP *per capita*l (CNY 80,976/QALY).

**Figure 5:**
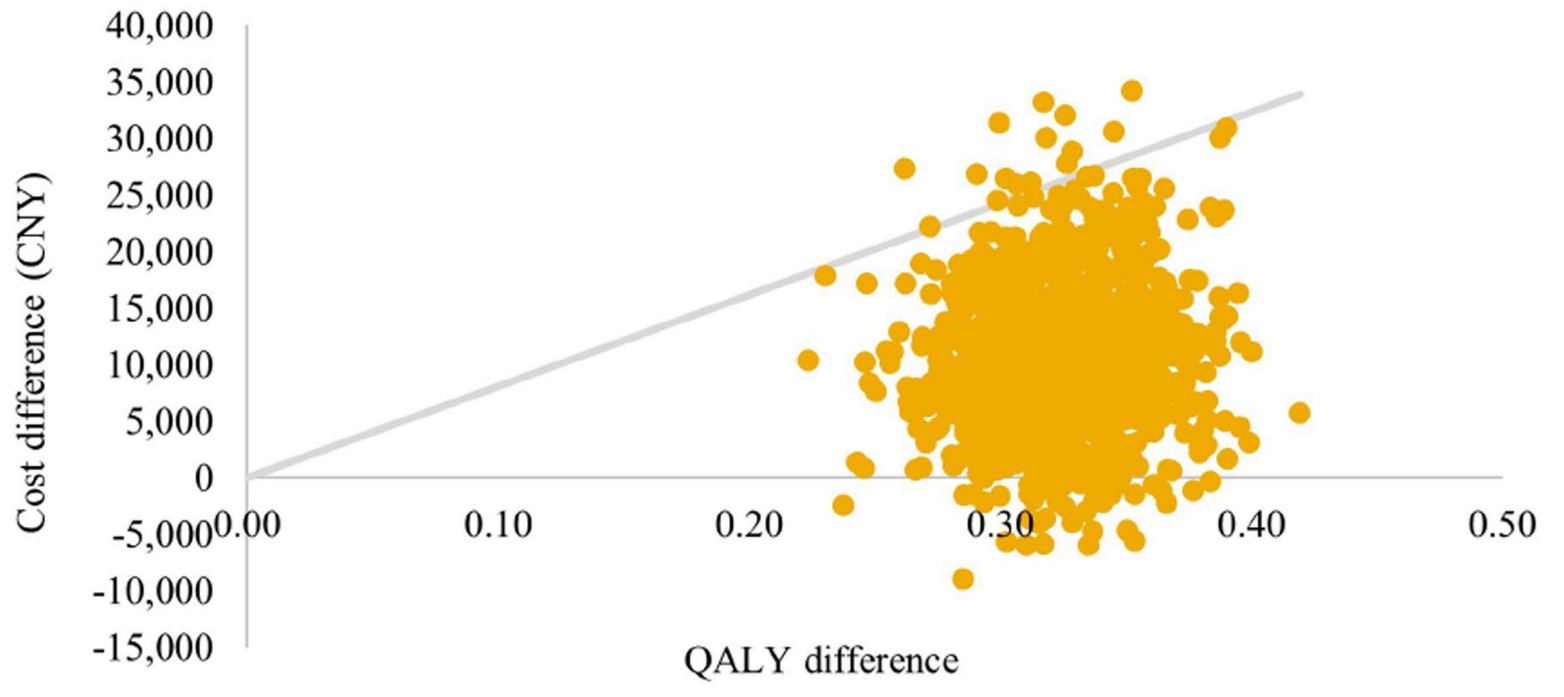
Cost-effectiveness plane: CKD cohort. QALYs, quality-adjusted life-years; CKD, chronic kidney disease.

**Figure 6:**
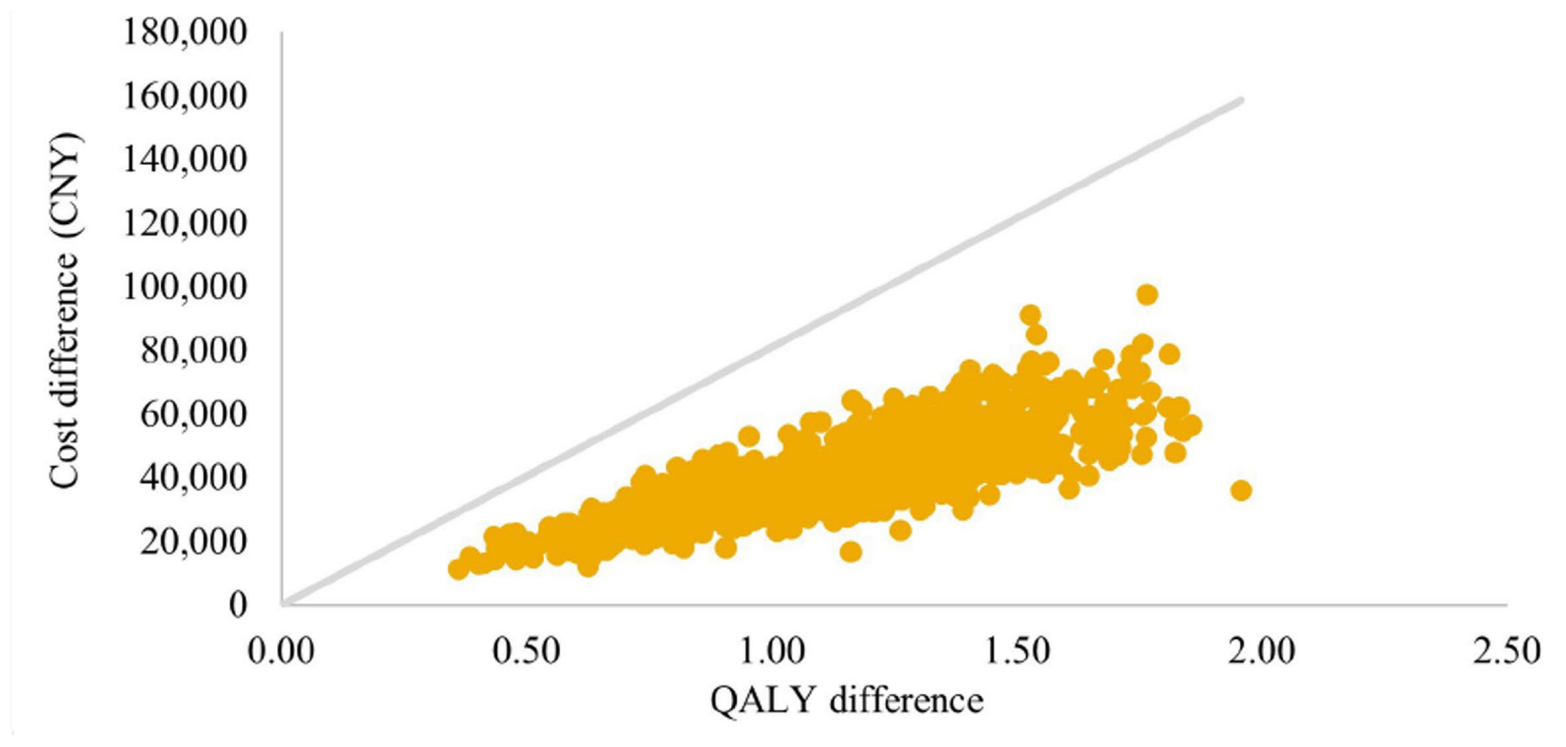
Cost-effectiveness plane: HF cohort. QALYs, quality-adjusted life-years; HF, heart failure.

Incremental differences in costs and QALYs between arms for 1,000 simulations are presented in a cost-effectiveness plane (Figures 5, 6). Under the strictest WTP threshold of 1 time GDP per capital (CNY 80,976 per QALY), the probability of SZC being cost-effective compared to usual care was 98.5% in CKD cohort and 100% in HF cohort.

## 4 Discussion

We modeled the potential clinical and economic impact of using SZC to treat hyperkalemia among CKD patients and HF patients and found SZC could be considered cost-effective compared with usual care. The model yielded improvements in survival and health-related quality of life and reductions in hyperkalemia events, hospitalization and cardiovascular events, which partially offset the cost of SZC. Using a willingness-to pay-threshold of CNY 80,976 per additional QALY gained as our threshold, the SZC was a cost-effective strategy across all of our sensitivity analyses, even the ICERs were less than the 1 time GDP per capita (CNY 80,976).

When used in effective doses to treat HF, using RAASi can reduce morbidity and mortality, but may raise serum potassium. RAASi can slow the progression of CKD, but it also increases the risk of serum potassium elevation. Therefore, in the simulation of the CKD cohort, we set different annual rates of eGFR decline according to whether patients received RAASi or not, the rate of eGFR decline was slower in patients who used RAASi. When serum potassium was higher than a set value, a certain proportion of patients in both arms would be down-titrated or discontinued RAASi. Though stabilizing serum potassium at a lower level, SZC reduces RAASi down-titration or discontinuation, thereby delaying CKD progression.

The results of placebo-controlled trial showed adverse events were comparable between SZC and placebo, and no AEs were considered related to SZC; the single-arm trial showed that in the acute correction phase, the incidence of all adverse events was only 4%, and in the long-term management phase, the incidence of all serious adverse events was less than 2%. Therefore, AE was not included in this model for the purpose of model simplification.

SZC, as a long-term management of hyperkalemia, increases the treatment duration, which is bound to result in increased costs, and because the annual costs were considered in this study, the increase of survival years in the SZC arm increased the total cost. On the other hand, SZC can reduce the incidence of each event by controlling serum potassium, thus reducing the cost of related events.

SZC provides a new option for the long-term management of hyperkalemia in China, which may help to construct a treatment modality for the long-term management of serum potassium, thereby preventing the occurrence of related events caused by elevated serum potassium level.

The cost-effectiveness analysis, however, has some limitations. First, due to the clinical trials of SZC are only placebo-controlled and single-arm design, the clinical efficacy data used in the study were derived from the results of ZS-004 and ZS-005 trials where few Chinese patients were included, we assumed that usual care were as effective as SZC in the acute phase conservatively. In addition, part of the baseline information of the population in the current study comes from international clinical trial studies, which may be different from the baseline of the Chinese population. Consequently, considering the ethnic differences, the applicability of clinical efficacy and baseline parameters to the Chinese population requires further discussion and analysis.

Second, since some cost parameters are unavailable from literature reviews or government publications, we conduct a survey to elicit clinical expert opinion to fill evidence gaps. In the actual clinical consultation process, the patient’s behavior of attendance and treatment may be more complicated. Although deterministic sensitivity analysis and probabilistic sensitivity analysis have been performed in the model, evidence from real-world data would be required to demonstrate robustness and extrapolation of the results.

Third, since this study was conducted from the perspective of the Chinese health care system, we did not consider irrelevant medical and opportunity costs.

In terms of utility, utility values were derived from a study in South Korea due to the lack of utility studies based on different grades of CKD population in China. However, the baseline information of affected individuals may be different, and the social conditions and cultural backgrounds of the two countries may affect the people’s cognition of utility. Thus the utility value currently used in the model may be different from the actual situation and may be replaced in the future if more appropriate utility values are available. An additional limitation is that this study did not consider the utility decline of acute hyperkalemia events, which occurred much less frequently in the SZC arm than in the usual care arm. Therefore, results are likely to underestimate the QALYs associated with the hyperkalemia events in SZC arm. This was made as a simplifying assumption because of the lack of disutility inputs.

## 5 Conclusion

We performed a cost-effectiveness analysis to evaluate the potential costs and health outcomes of using SZC and usual care to treat hyperkalemia among Chinese CKD and HF patients. This study suggests that SZC is likely to be considered cost-effective from the perspective of the Chinese healthcare system when compared with usual care, and these findings were robust to sensitivity analyses.

## Data availability statement

The original contributions presented in the study are included in the article/Supplementary material, further inquiries can be directed to the corresponding author.

## Author contributions

LT, SF, and HL: concept and design. SF, ML, and XZ: data acquisition. LT, SF, ML, and XZ: development of decision analytical model. SF and ML: data analysis. All authors contributed to the article and approved the submitted version.
