# Supplementary material for: Cost-effectiveness analysis of sodium zirconium cyclosilicate for treating hyperkalemia among Chinese patients
Source: Front Public Health. 2023 Dec 7;11:1196789. doi: 10.3389/fpubh.2023.1196789 (PMC10740179; doi:10.3389/fpubh.2023.1196789)
Supplement: Supplementary file 1 [file Table_1.docx]

Supplementary Material

Cost-effectiveness Analysis of Sodium Zirconium Cyclosilicate for Treating Hyperkalemia among Chinese patients

**Lei Tian ^1^, Shihui Fu ^1^, Mengyuan Li ^1^, Xinrui Zhao ^1^, Hongchao Li ^1^***

^1^ School of International Pharmaceutical Business, China Pharmaceutical University, Nanjing, China

*** Correspondence:** Hongchao Li: [lihongchao@cpu.edu.cn](mailto:lihongchao@cpu.edu.cn)

# Supplementary Material

**Supplementary Table S1.** Monthly transition probability of patients with heart failure

|  | NHYA I | NYHA II | NYHA III | NYHA IV |
| --- | --- | --- | --- | --- |
| NHYA I | 0.7956 | 0.1245 | 0.0738 | 0.0061 |
| NYHA II | 0.071 | 0.8448 | 0.0765 | 0.0077 |
| NYHA III | 0.0047 | 0.0893 | 0.8845 | 0.0216 |
| NYHA IV | 0 | 0.1064 | 0.1064 | 0.7872 |

**Supplementary** **Table S2.** Mixed-effects model parameters of modelling serum K+

|  | Model/term | Day ＜3 | Day 4-14 | Day 15-28 | Day >28 |
| --- | --- | --- | --- | --- | --- |
| Baseline K+：5.5 mmol/L | | | | | |
| Control arm | Fixed: Intercept | 5.965 | 5.084 | 5.355 | 5.355 |
|  | Fixed: Time (days) | -0.435 | - | - | - |
|  | Random: Patient | 0.275 | 0.300 | 0.300 | 0.300 |
|  | Random: Observation | 0.336 | 0.410 | 0.410 | 0.410 |
| Intervention arm | Fixed: Intercept | 5.965 | 4.808 | 4.808 | 4.692 |
|  | Fixed: Time (days) | -0.435 | - | - | - |
|  | Random: Patient | 0.275 | 0.340 | 0.340 | 0.340 |
|  | Random: Observation | 0.336 | 0.412 | 0.412 | 0.412 |
| Baseline K+：6.0 mmol/L | | | | | |
| Control arm | Fixed: Intercept | 6.468 | 5.329 | 5.478 | 5.478 |
|  | Fixed: Time (days) | -0.536 | - | - | - |
|  | Random: Patient | 0.234 | 0.164 | 0.164 | 0.164 |
|  | Random: Observation | 0.378 | 0.501 | 0.501 | 0.501 |
| Intervention arm | Fixed: Intercept | 5.600 | 5.077 | 4.880 | 4.770 |
|  | Fixed: Time (days) | -0.150 | - | - | - |
|  | Random: Patient | 0.237 | 0.216 | 0.216 | 0.216 |
|  | Random: Observation | 0.240 | 0.238 | 0.238 | 0.238 |

**Supplementary Table S3**. RAASi dose titration parameters

| Input parameters | Values | Distributions | SE | Sources |
| --- | --- | --- | --- | --- |
| **Proportion of patients discontinuing** | | | | |
| From maximum RAASi dose: K+ < 5.1 | 0 | Beta | 0 | Epstein et al., 2015^[2]^ |
| From maximum RAASi dose: K+ 5.1-5.4 | 0.244 | Beta | 0.003 | Epstein et al., 2015^[2]^ |
| From maximum RAASi dose: K+ ≥5.5 | 0.295 | Beta | 0.004 | Epstein et al., 2015^[2]^ |
| From sub-maximum RAASi dose: K+ < 5.1 | 0 | Beta | 0 | Epstein et al., 2015^[2]^ |
| From sub-maximum RAASi dose: K+ 5.1-5.4 | 0.282 | Beta | 0.002 | Epstein et al., 2015^[2]^ |
| From sub-maximum RAASi dose: K+ ≥5.5 | 0.329 | Beta | 0.002 | Epstein et al., 2015^[2]^ |
| **Proportion of patients down-titrating** | | | | |
| From maximum RAASi dose: K+ < 5.1 | 0 | Beta | 0 | Epstein et al., 2015^[2]^ |
| From maximum RAASi dose: K+ 5.1-5.4 | 0.178 | Beta | 0.002 | Epstein et al., 2015^[2]^ |
| From maximum RAASi dose: K+ ≥5.5 | 0.239 | Beta | 0.004 | Epstein et al., 2015^[2]^ |
| **Proportion of patients returning to maximum dose** | | | | |
| Discontinuation | 0.497 | Normal | 0.050 | Luo et al, 2016^[3]^ |
| Down-titration | 0.497 | Normal | 0.050 | assumption |
| **If return, time to maximum dose (weeks)** | | | | |
| Discontinuation | 42.286 | Normal | 4.229 | Luo et al, 2016^[3]^ |
| Down-titration | 42.286 | Normal | 4.229 | assumption^[3]^ |

RAASi: renin-angiotensin-aldosterone system inhibitors

**Supplementary Table S4** IRR for hospitalization, cardiovascular events and death, by serum K+ level[3]

| IRR  mean (SE*) | | Serum K+ subgroup (mmol/L) | | | | | | |
| --- | --- | --- | --- | --- | --- | --- | --- | --- |
|  |  | <3.5 | 3.5-3.9 | 4-4.4 | 4.5-4.9 | 5-5.4 | 5.5-5.9 | ≥6 |
| hospitalisation | eGFR <30 | 1.93 (0.45) | 1.65 (0.22) | 0.93 (0.09) | 1 | 1  (0.10) | 1.34 (0.18) | 3.65 (0.58) |
|  | eGFR 30-40 | 1.77 (0.34) | 1.35 (0.16) | 0.99 (0.08) | 1 | 0.96 (0.09) | 1.07 (0.14) | 1.82 (0.44) |
|  | eGFR 40-50 | 2.24 (0.38) | 1.23 (0.13) | 1.08 (0.07) | 1 | 1.07 (0.09) | 1.23 (0.18) | 1.91 (0.56) |
|  | eGFR 50-60 | 2.06 (0.27) | 1.13 (0.08) | 1.01 (0.05) | 1 | 1  (0.07) | 0.81 (0.11) | 1.07 (0.30) |
| cardiovascular events | | 1.89 (0.09) | 1.27 (0.04) | 1.04 (0.01) | 1 | 1.01 (0.02) | 1.12 (0.04) | 1.88 (0.12) |
| death | | 3.05 (0.29) | 1.49 (0.08) | 1.06 (0.04) | 1 | 1.14 (0.06) | 1.6 (0.13) | 3.31 (0.46) |

*SE estimated from 95% confidence interval

IRR: Incidence rate ratio; CKD: chronic kidney disease; eGFR: estimated glomerular filtration rate

**Supplementary Table S5.** The Seattle Heart Failure Model[4]

| Variable | HR | 95% CI | SE |
| --- | --- | --- | --- |
| λ | -0.0405 |  | 0 |
| If hemoglobin <16, 16-hemoglobin (g/dl) | 1.124 | (1.053–1.200) | 0.0375 |
| If hemoglobin >16, hemoglobin -16 (g/dl) | 1.336 | (1.010–1.767) | 0.1931 |
| If sodium ion <138(mmol/L) | 1.050 | (1.005–1.097) | 0.0235 |
| Age (year /10) | 1.090 | (0.985–1.205) | 0.0561 |
| male | 1.089 | (0.839–1.414) | 0.1467 |
| NYHA (1–4) | 1.600 | (1.019–2.511) | 0.3806 |
| 100/ ejection fraction | 1.030 | (1.010–1.050) | 0.0102 |
| Ischemic cause (0/1) | 1.354 | (1.074–1.707) | 0.1615 |
| SBP (mmHg/10) | 0.877 | (0.823–0.935) | 0.0286 |
| Diuretic dose (mg/kg) | 1.178 | (1.097–1.266) | 0.0431 |
| Allopurinol use (0/1) | 1.571 | (1.170–2.109) | 0.2395 |
| Statin use (0/1) | 0.630 | (0.410–0.978) | 0.1449 |
| Cholesterol (100/mg/dL) | 2.206 | (1.045–4.656) | 0.9212 |
| Lymphocyte (%/5) | 0.897 | (0.846–0.951) | 0.0523 |
| Uric acid (mg/dL) | 1.064 | (1.022–1.108) | 0.0219 |
| ACEI use (0/1) | 0.770 | - | - |
| Beta blocker use (0/1) | 0.660 | - | - |
| ARB use (0/1) | 0.850 | - | - |
| Potassium-preserving diuretic use (0/1) | 0.740 | - | - |
| Implantable cardioverter Defibrillator (0/1) | 0.730 | - | - |
| Biventricular implanted cardioverter defibrillator (0/1) | 0.790 | - | - |
| The annual death probability of patients with heart failure can be calculated based on baseline data and parameters in the heart failure model. Annual mortality rates were calculated based on different serum potassium levels and converted to the probability of death per cycle.  SHFM Score = ((age 10) *Ln (1.09)) + (gender (male) * Ln (1.089)) + (NYHA * Ln (1.6)) + ((100 / ejection fraction) *Ln (1.03)) + (ischemic etiology * Ln (1.354)) + ((SBP / 10) *Ln (0.877)) + ((diuretic dose/kg) * Ln (1.178)) + (allopurinol * Ln (1.571)) + (statin * Ln (0.63)) + (sodium * Ln (1.05)) + ((100 / cholesterol) * Ln(2.206)) + ((16 - hemoglobin) * Ln (1.124)) + ( (lymphocytes % / 5) * Ln (0.897)) + (uric acid * Ln (1.064)) + (ACEI * Ln (0.77)) + (beta * Ln (0.66)) + (ARB * Ln (0.85)) + (potassium diuretics * Ln (0.74)) + (ICD * Ln (0.73)) + (BICD * Ln (0.79))  Survival = e(− λ*t)e^(SHFM Score)^ t = time ， λ =0.0405  P = 1 – Survival  r = - Ln (1 - P)*HR*（4/52）  P=1 - e^(-r)^ | | | |

ACE: angiotensin converting enzyme; ARB: angiotensin II receptor blocker; BICD: biventricular implantable cardioverter defibrillator; NYHA: New York Heart Association; RAASi: renin-angiotensin-aldosterone system inhibitor; SHFM: Seattle Heart Failure Model.

**Supplementary Table S6.**  Additional baseline characteristic for patients with heart failure

| Characteristic | Mean | Reference |
| --- | --- | --- |
| Ejection fraction (mL) | 48.00 | Zhang et al, 2017 ^[5]^ |
| Ischemic etiology | 0.62 | MacDonald et al, 2020 ^[6]^ |
| Medical history: diabetes mellitus | 0.20 | Zhang et al, 2017 ^[5]^ |
| History: Cancer | 0.04 | Wang et al, 2019 ^[7]^ |
| History: Metastatic tumor | 0.00 | Assumption |
| History: Peripheral vascular disease | 0.09 | Yu et al, 2019 ^[8]^ |
| History: Dementia | 0.00 | Assumption |
| History: Cardiovascular events | 0.18 | Zhang et al, 2017 ^[6]^ |
| History: Rheumatism | 0.00 | Assumption |
| History: CPD | 0.08 | Wang et al, 2019 ^[7]^ |
| Smoking | 0.23 | Wang et al, 2019 ^[7]^ |
| Body weight (kg) | 60.00 | Yu et al, 2019 ^[8]^ |
| BMI (kg/m^2^) | 23.70 | Zhang et al, 2017 ^[5]^ |
| Systolic blood pressure (mmHg) | 128.00 | Zhang et al, 2017 ^[5]^ |
| Cholesterol (mg/dL) | 231.63 | Wang et al, 2019 ^[7]^ |
| Hemoglobin (g/dL) | 12.90 | Wang et al, 2019 ^[7]^ |
| White blood cell count (x10^9^/L) | 7.56 | Techinical ducument ^[9]^ |
| Lymphocyte (10^3^ cells/µL) | 2.15 | Techinical ducument ^[9]^ |
| Blood sodium (mmol/L) | 139.40 | Wang et al, 2019 ^[7]^ |
| Uric acid (mg/dL) | 7.00 | Wang et al, 2019 ^[7]^ |
| Drug use: diuretics | 0.30 | Zhang et al, 2017 ^[5]^ |
| Drug use: potassium-preserving diuretics | 0.00 | Assumption |
| Drug use: beta blockers | 0.26 | Zhang et al, 2017 ^[5]^ |
| Drug use: Calcium channel blockers | 0.07 | Zhang et al, 2017 ^[5]^ |
| Drug use: islets | 1.00 | Zhang et al, 2017 ^[5]^ |
| Drug use: Statins | 0.00 | assumption |
| Drug use: Bronchodilators | 0.23 | Wang et al, 2019 ^[7]^ |
| Drug use: allopurinol | 0.00 | assumption |
| Drug use: ICDs | 0.01 | Wang et al, 2019 ^[7]^ |
| Drug use: BICD | 0.00 | assumption |
| Proportion of ACEI in patients receiving RAASi | 0.62 | Zhang et al, 2017 ^[5]^ |
| Proportion of ARBs in patients receiving RAASi | 0.39 | Zhang et al, 2017 ^[5]^ |
| Diuretic dose (mg/kg) | 1.33 | Chinese Guidelines^[10]^ |
| Proportion of NYHA I at baseline | 23% | Jackson et al, 2018 ^[11]^ |
| Proportion of NYHA II at baseline | 48% | Jackson et al, 2018 ^[11]^ |
| Proportion of NYHA III at baseline | 25% | Jackson et al, 2018 ^[11]^ |
| Proportion of NYHA IV at baseline | 4% | Jackson et al, 2018 ^[11]^ |
| The baseline information of the heart failure population was based on the international large clinical trial data (ZS-005 and ZS-004) on the treatment of hyperkalemia with sodium zirconium cyclosilicate, and other baseline characteristics were obtained from the relevant studies on the heart failure population. If the disease history and the proportion of medication in the baseline condition could not be found, 0 was assumed. | | |

ACE: angiotensin converting enzyme; ARB: angiotensin II receptor blocker; BICD: biventricular implantable cardioverter defibrillator, BMI: body mass index; CPD: chronic pulmonary disease; ICD: implantable cardioverter defibrillator; NYHA: New York Heart Association; RAASi: renin-angiotensin-aldosterone system inhibitor.

**Supplementary Table S7.** HR of death in patients with heart failure, by serum K+ levels [12]

| Serum K+ subgroup (mmol/L) | <3.5 | 3.5-3.9 | Standard 3.9-4.3 | 4.3-4.6 | 4.6-5.1 | 5.1-5.5 | >5.5 |
| --- | --- | --- | --- | --- | --- | --- | --- |
| hazard ratio of death | 2.19 | 1.91 | 1 | 1 | 1.47 | 2.28 | 6.60 |

**Supplementary Table S8.** Risk equation for calculating the incidence of cardiovascular events in patients with HF[13]

| Variable | Estimate | SE | *t* statistic | P-value |
| --- | --- | --- | --- | --- |
| Constant | -3.1193 | 0.2433 | -12.8220 | <0.0001 |
| Serum potassium: <3.5 mmol/L | 0.1331 | 0.0970 | 1.3723 | 0.1554 |
| Serum potassium: 3.5 to <4.0 mmol/L | 0.1382 | 0.0432 | 3.1982 | 0.0024 |
| Serum potassium: 4.0 to <4.5 mmol/L | 0.0701 | 0.0336 | 2.0872 | *0.0455* |
| Serum potassium: 5.0 to <5.5 mmol/L | 0.0080 | 0.0487 | 0.3935 | *0.3942* |
| Serum potassium: 5.5 to <6.0 mmol/L | -0.0147 | 0.1145 | -0.1284 | *0.3956* |
| Serum potassium: ≥6.0 mmol/L | 0.1297 | 0.2512 | 0.5162 | *0.3492* |
| Age at baseline (years) | 0.0144 | 0.0013 | 11.3253 | <0.0001 |
| Gender at baseline: Female | -0.0775 | 0.0315 | -2.4616 | 0.0193 |
| Time with HF (days) | -0.0005 | 0.0000 | -14.8884 | <0.0001 |
| History of cardiovascular events at baseline: Yes | 0.3100 | 0.0311 | 9.9799 | <0.0001 |
| History of cancer at baseline: Yes | 0.1475 | 0.0486 | 3.0342 | 0.0040 |
| History of PVD at baseline: Yes | 0.2741 | 0.0767 | 3.5724 | 0.0007 |
| Natural logarithm of baseline white blood cell count (x10^9^/L) | 0.1856 | 0.1002 | 1.8510 | 0.0782 |
| Prescribed diuretics ± 3 months of baseline: Yes | 0.5274 | 0.0368 | 14.3317 | <0.0001 |
| Prescribed beta blockers ± 3 months of baseline: Yes | 0.2248 | 0.0342 | 6.5690 | <0.0001 |
| The probability of cardiovascular events per cycle in patients with heart failure can be calculated based on baseline patient data and parameters in the equation:  Sum = ln (4/52) -3.1193 + (age * 0.0144) + (sex (female) * -0.0775) + (time of diagnosis * -0.0005) + (history of cardiovascular events * 0.3100) + (cancer * 0.1475) + (PVD * 0.2741) + (ln[WBC] * 0.1856) + (Diuretic * 0.5274) + (beta-blocker * 0.2248)  If K+ < 3.5, Sum = Sum + (1 * 0.1331)  If K+ ≥3.5 and <4, Sum = Sum + (1 * 0.1382)  If K+ ≥4, <4.5, Sum = Sum + (1 * 0.0701)  If K+ ≥4.5 Sum = Sum  r = e ^Sum^  p = 1 – e(-r) | | | | |

HF: heart failure; PVD: peripheral vascular disease; SE: standard error.

**Supplementary Table S9.** Probability of hospitalization in patients with HF, by serum K+ level and RAASi dose[13,14]

| probability of hospitalization in patients with HF | Mean |
| --- | --- |
| maximum RAASi dose, ＜5.5mmol/L | 0.067 |
| maximum RAASi dose, 5.5-6.0 mmol/L | 0.083 |
| maximum RAASi dose, ≥6.0mmol/L | 0.117 |
| sub-maximal RAASi dose, ＜5.5mmol/L | 0.081 |
| sub-maximal RAASi dose, 5.5-6.0 mmol/L | 0.091 |
| sub-maximal RAASi dose, ≥6.0mmol/L | 0.114 |
| no RAASi, ＜5.5mmol/L | 0.105 |
| no RAASi, 5.5-6.0 mmol/L | 0.105 |
| no RAASi, ≥6.0mmol/L | 0.110 |

**Reference**

1. Yao G, Freemantle N, Calvert M J, et al. The long-term cost-effectiveness of cardiac resynchronization therapy with or without an implantable cardioverter-defibrillator[J]. European Heart Journal, 2006,28(1):42-51.

2. Epstein M, Reaven N L, Funk S E, et al. Evaluation of the treatment gap between clinical guidelines and the utilization of renin-angiotensin-aldosterone system inhibitors[J]. Am J Manag Care, 2015,21(11 Suppl):S212-S220.

3. Luo J, Brunelli S M, Jensen D E, et al. Association between Serum Potassium and Outcomes in Patients with Reduced Kidney Function[J]. Clin J Am Soc Nephrol, 2016,11(1):90-100.

4. Levy W C, Mozaffarian D, Linker D T, et al. The Seattle Heart Failure Model[J]. Circulation, 2006,113(11):1424-1433

5. Zhang Y, Zhang J, Butler J, et al. Contemporary Epidemiology, Management, and Outcomes of Patients Hospitalized for Heart Failure in China: Results From the China Heart Failure (China-HF) Registry[J]. Journal of Cardiac Failure, 2017,23(12):868-875.

6. MacDonald M R, Tay W T, Teng T H K, et al. Regional Variation of Mortality in Heart Failure With Reduced and Preserved Ejection Fraction Across Asia: Outcomes in the ASIAN‐HF Registry[J]. Journal of the American Heart Association, 2020,9(1).

7. Wang Hua, Li Yingying, Chai Ke, et al. Epidemiology and treatment of hospitalized patients with heart failure in China [J]. Chinese Journal of Cardiovascular Diseases, 2019,47(11):865-874. (in Chinese)

8. Yu Y, Gupta A, Wu C, et al. Characteristics, Management, and Outcomes of Patients Hospitalized for Heart Failure in China: The China PEACE Retrospective Heart Failure Study[J]. Journal of the American Heart Association, 2019,8(17).

9. AstraZeneca Corp. Techinical ducument. Lokelma (ZS) cost-effectiveness model for the management of hyperkalaemia in CKD and HF patients[Z]. 2018.

10. Heart failure Group, Cardiology Branch of Chinese Medical Association, Heart failure Professional Committee of Chinese Medical Doctor Association, Editorial Board of Chinese Journal of Cardiovascular Diseases. Chinese Guidelines for diagnosis and Treatment of heart failure 2018[J]. Chinese Journal of Heart Failure and Cardiomyopathy, 2018,2(4).

11. Jackson J D, Cotton S E, Wirta S B, et al. Burden of heart failure on patients from China: results from a cross-sectional survey[J]. Drug Des Devel Ther, 2018,12:1659-1668.

12. Krogager M L, Eggers-Kaas L, Aasbjerg K, et al. Short-term mortality risk of serum potassium levels in acute heart failure following myocardial infarction[J]. European Heart Journal - Cardiovascular Pharmacotherapy, 2015,1(4):245-251.

13. Linde C, Qin L, Bakhai A, Furuland H, Evans M, Ayoubkhani D, Palaka E, Bennett H, McEwan P. Serum potassium and clinical outcomes in heart failure patients: results of risk calculations in 21 334 patients in the UK. ESC Heart Fail. 2019 Apr;6(2):280-290.

14. Packer M, Poole-Wilson P, Armstrong P, et al. Clinical Investigation and Reports Comparative Effects of Low and High Doses of the Angiotensin-Converting Enzyme Inhibitor, Lisinopril, on Morbidity and Mortality in Chronic Heart Failure[J]. Circulation, 1999,100(23):2312-2318.
